# Publisher Correction: *Solanum americanum* genome-assisted discovery of immune receptors that detect potato late blight pathogen effectors

**DOI:** 10.1038/s41588-023-01550-4

**Published:** 2023-09-25

**Authors:** Xiao Lin, Yuxin Jia, Robert Heal, Maxim Prokchorchik, Maria Sindalovskaya, Andrea Olave-Achury, Moffat Makechemu, Sebastian Fairhead, Azka Noureen, Jung Heo, Kamil Witek, Matthew Smoker, Jodie Taylor, Ram-Krishna Shrestha, Yoonyoung Lee, Chunzhi Zhang, Soon Ju Park, Kee Hoon Sohn, Sanwen Huang, Jonathan D. G. Jones

**Affiliations:** 1 The Sainsbury Laboratory, University of East Anglia, Norwich Research Park, Norwich, UK; 2 State Key Laboratory of Plant Genomics, Institute of Microbiology, Chinese Academy of Sciences, Beijing, China; 3 Shenzhen Branch, Guangdong Laboratory of Lingnan Modern Agriculture, Genome Analysis Laboratory of the Ministry of Agriculture and Rural Area, Agricultural Genomics Institute at Shenzhen, Chinese Academy of Agricultural Sciences, Shenzhen, China; 4 Key Laboratory for Potato Biology of Yunnan Province, The CAAS-YNNU-YINMORE Joint Academy of Potato Science, Yunnan Normal University, Kunming, China; 5 Department of Life Sciences, Pohang University of Science and Technology, Pohang, Republic of Korea; 6 Department of Biological Science and Institute of Basic Science, Wonkwang University, Iksan, Republic of Korea; 7 Division of Applied Life Sciences and Plant Molecular Biology and Biotechnology Research Center (PMBBRC), Gyeongsang National University, Jinju, Republic of Korea; 8 School of Interdisciplinary Bioscience and Bioengineering, Pohang University of Science and Technology, Pohang, Republic of Korea; 9 Department of Agricultural Biotechnology, Seoul National University, Seoul, Republic of Korea; 10 Plant Immunity Research Center, Seoul National University, Seoul, Republic of Korea; 11 Present Address: Plant Pathology Group, The Institute of Crop Science and Resource Conservation, University of Bonn, Bonn, Germany

**Keywords:** Plant genetics, Genomics

Correction to: *Nature Genetics* 10.1038/s41588-023-01486-9, published online 28 August 2023.

In the version of this article originally published, there was a typographical error in the Table 1 “Total assembly” entry for “SP2273,” where the assembly size now reading “1.02 Gb” appeared initially as “0.02 Gb”. The error is now corrected in the HTML and PDF versions of the article.

