# Supplementary material for: Solanum americanum genome-assisted discovery of immune receptors that detect potato late blight pathogen effectors
Source: Nat Genet. 2023 Aug 28;55(9):1579–88. doi: 10.1038/s41588-023-01486-9 (PMC10484786; doi:10.1038/s41588-023-01486-9)
Supplement: Supplementary file 1 — Supplementary Notes 1–3 and Figs. 1–19. [file 41588_2023_1486_MOESM1_ESM.pdf]

nature genetics

Article https://doi.org/10.1038/s41588-023-01486-9

Solanum americanum genome-assisted discovery of immune receptors that detect potato late blight pathogen effectors

In the format provided by the authors and unedited

Supplementary Note 1. Genome assembly of the four *S. americanum* accessions

We first generated Illumina paired-end reads to analyze these genomes. K-mer based analysis indicated that the genome sizes of these four *S. americanum* accessions were 1.15-1.31 Gb, with 0.05% to 0.35% heterozygosity (Table 1, Supplementary Fig. 2). We then generated an average of 29.5 Gb (~26 fold) PacBio high-fidelity reads for SP1102 and SP2271, and assembled the reads into 298 and 568 contigs with N50s 82.9 Mb and 55.2 Mb, respectively. The ONT platform was used for sequencing SP2273 and SP2275, resulting in ~81.1 Gb (~62 fold) and ~114.5 Gb of ONT reads (~95 fold), respectively. The noisy reads were self-corrected and assembled into 304 and 539 contigs with N50s 9.6 Mb and 4.8 Mb, respectively. To generate chromosome level assemblies, we further generated ~86.5 Gb, ~81.8 Gb and ~54.8 Gb Hi-C data for SP1102, SP2271 and SP2273, and anchored the contigs into 12 pseudomolecules (Table 1, Supplementary Fig. 3).

Supplementary Note 2. Structural variations among *S. americanum* genomes.

To further characterize small SVs (40 bp - 1 Mbp in size) within *S. americanum*, we used SP1102 as the reference and identified 60,849, 23,672 and 18,492 SVs in SP2271, SP2273 and SP2275, ranging from 27.9 to 102.0 Mb in total length (Supplementary Fig. 7). Most SVs (~54.5% on average) located in putative gene regulatory regions (5 kb upstream, downstream or in introns), followed by 44.0% and 1.5% overlapped with intergenic region and exon. SVs might contribute to gene expression variation[37]. We identified 1,837 differentially expressed genes (DEGs, Fold change ≥ 2 or ≤ 0.5, P-value < 0.05) in leaves between SP1102 and SP2271, of which, 1,084 DEGs might be associated with SVs. For example, the reduced expression of *sp1102chr11_nlr_6* in leaves of SP2271 might associated with the 286 bp deletion in its promoter region (Supplementary Fig. 8).

Supplementary Note 3. Pan-NLRome of *S. americanum*.

To comprehensively understand the *NLR* repertoire in *S. americanum*, we built a pan-NLRome based on *NLRs* from four genome assemblies and 16 SMRT RenSeq assemblies (Supplementary Fig. 9a). The total number of *NLR* orthogroups increased when incorporating additional accessions, and nearly approached a plateau when n = 19, which suggests that the accessions in our research are representative of *S. americanum NLR* repertoire. The number of core, dispensable and unique *NLR* genes from each accessions is shown (Supplementary Fig. 9b).

Supplemental Figures:

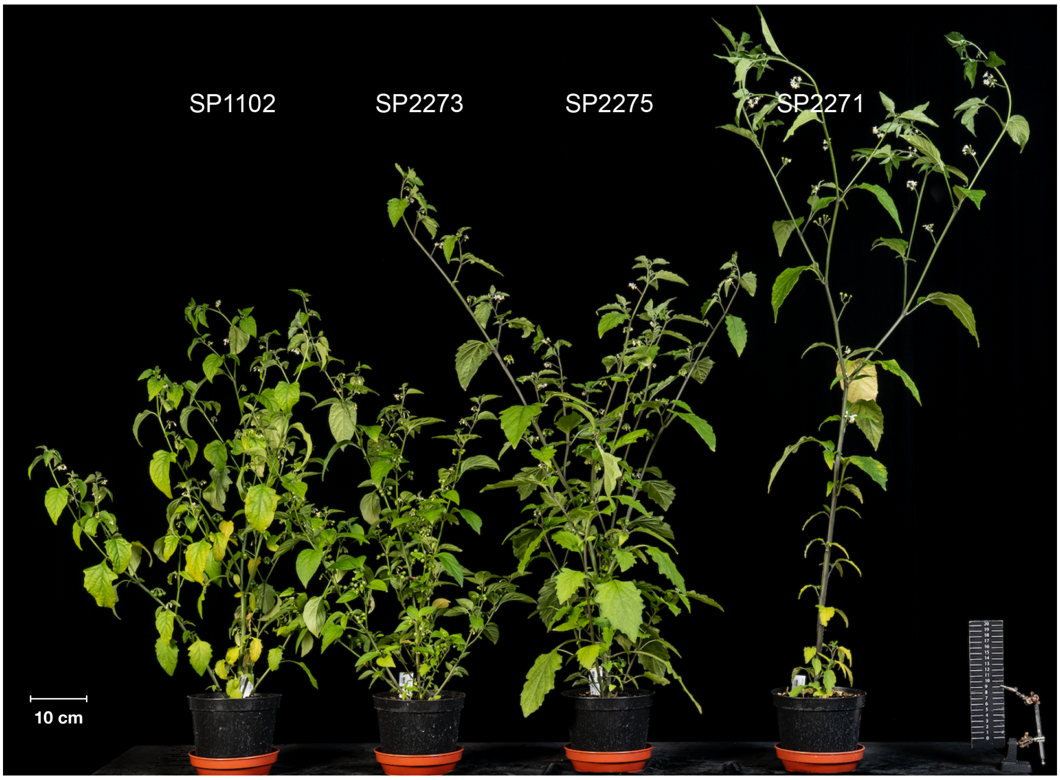

Supplementary Figure 1. (a). Phenotypes of four *S. americanum* accessions at the flowering stage, a ruler of 20 cm is shown. (b). The late blight resistance of *S. americanum*, SP1102, SP2273 and SP2275 are highly resistant to late blight, the susceptibility of SP2271 is age-dependent, the young leaves are more resistant to late blight, but the older leaves are more susceptible. Seven-eight weeks plants were used in this disease test, six leaves from top to bottom were detached and inoculated by *P. infestans* T30-4 (500 zoospores/droplet). *N. benthamiana* and *S.* tuberosum were included as controls. Three biological replicates were performed for SP2271, all datapoints (24 datapoints/leaf) were visualized as a box-and-whisker plot using R. Center line, median; box limits, upper and lower quartiles. The whiskers (top and bottom) comprise values within 1.5 times of the interquartile range (IQR). The outliers are indicated by black dots. Scar bar, 10 cm.

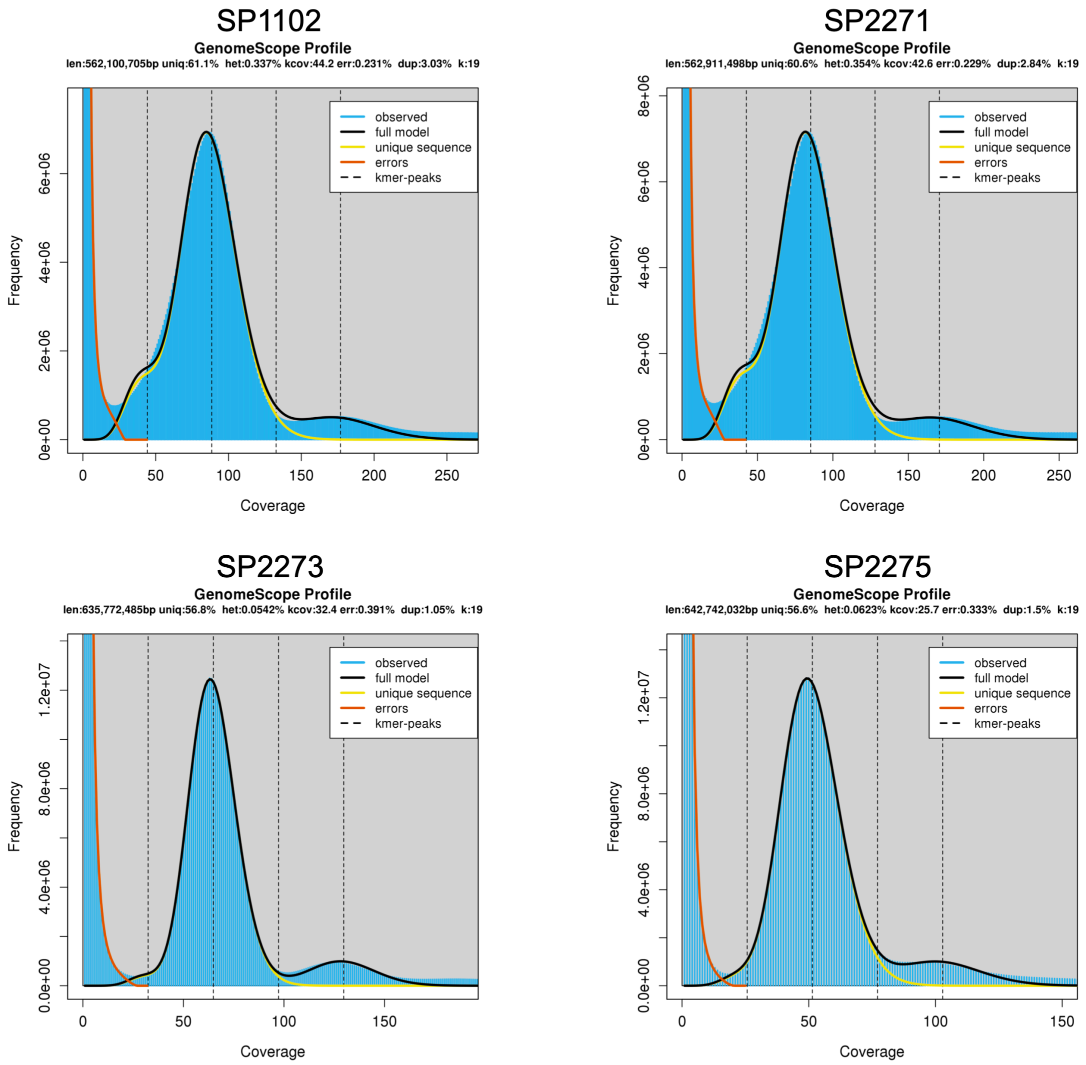

Supplementary Figure 2. K-mer frequency distribution based on Illumina reads of *S. americanum* genome.

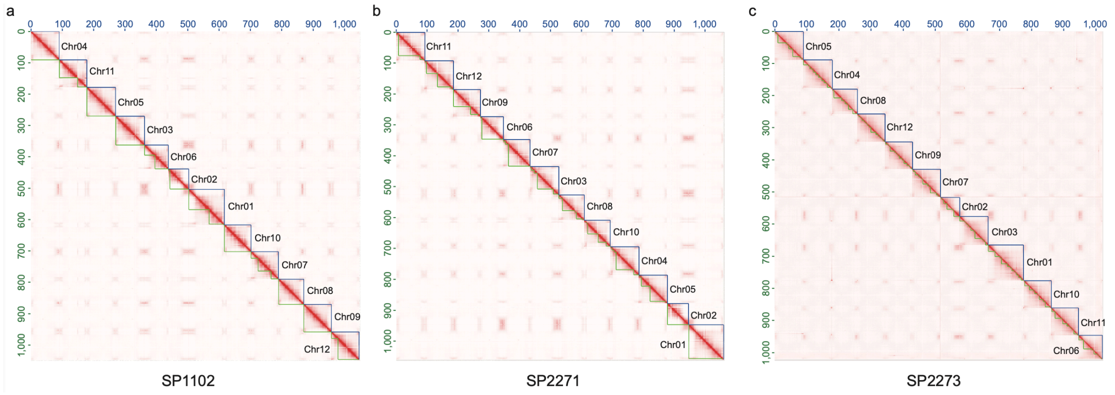

Supplementary Figure 3. Hi-C interaction maps of SP1102, SP2271 and SP2273. The maps were visualized by Juicebox with 2.5 Mb resolution. Blue box indicates pseudomolecules; Green box stands for contigs. For ease of inspection, only half of each box is shown in the figure.

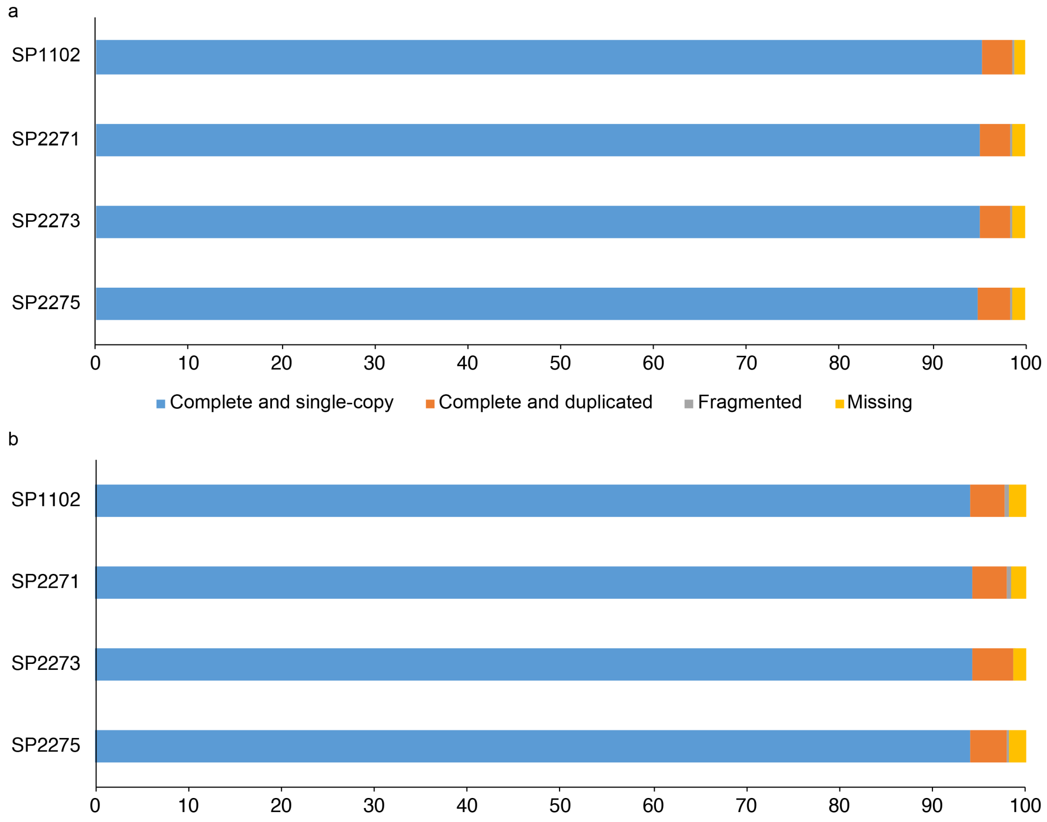

Supplementary Figure 4. BUSCO evaluation of *S. americanum* genome assemblies and gene model predictions. (a). BUSCO evaluation for genome assemblies of *S. americanum*. (b). The evaluation of *S. americanum* gene model prediction. The solanales_odb10 database was used for BUSCO evaluation.

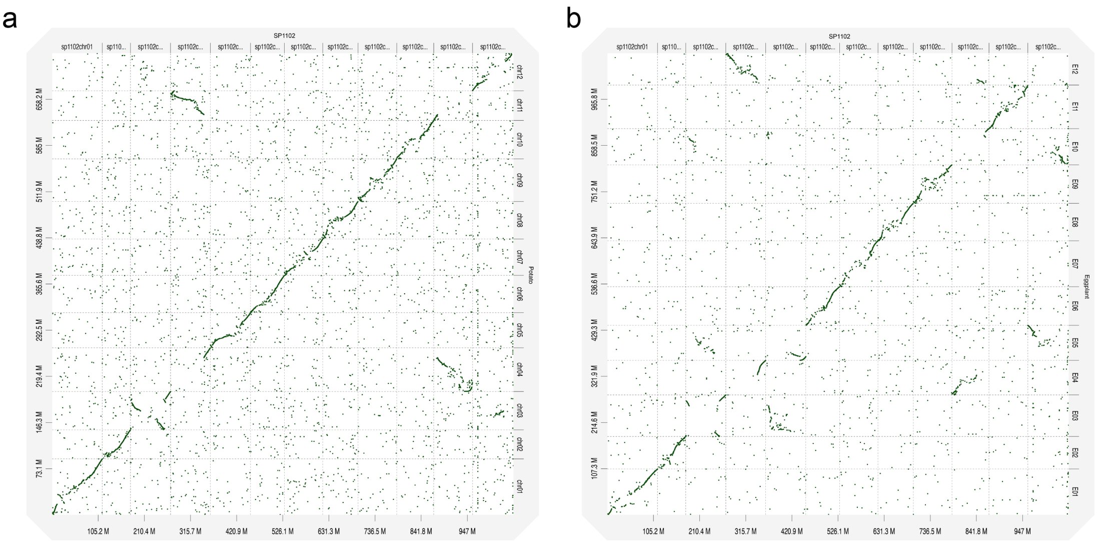

Supplementary Figure 5. Genome alignment between *S. americanum* and neighbouring species. (a). Genome alignment between *S. americanum* (SP1102) and potato (DM). (b). Genome alignment between *S. americanum* (SP1102) and eggplant (HQ1315). Each dot in the figure represents a pair-wise alignment, only alignments with length ⩾ 100 bp and identity ⩾ 80% were kept.

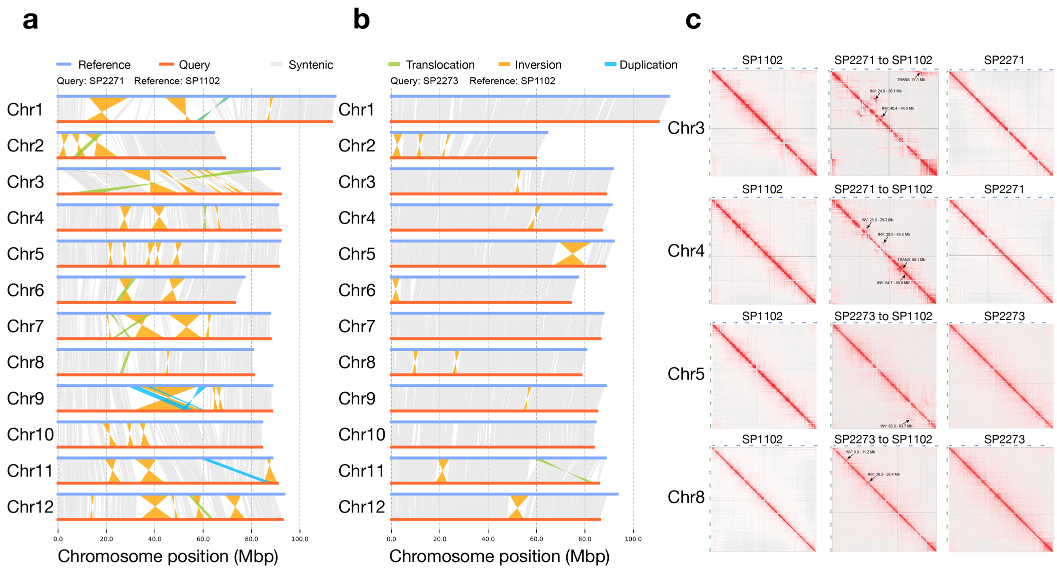

Supplementary Figure 6. Large structural variations (>1 Mb) between *S. americanum* genomes. (a). SP2271 vs SP1102. (b). SP2273 vs SP1102. (c). Example of SV validation by Hi-C interaction map. Four chromosomes were selected as example of verify large SVs by Hi-C data. The validation was performed by mapping the Hi-C reads to its corresponding genome or to SP1102 reference genome. Arrows indicate SVs that could be validated by Hi-C interaction map.

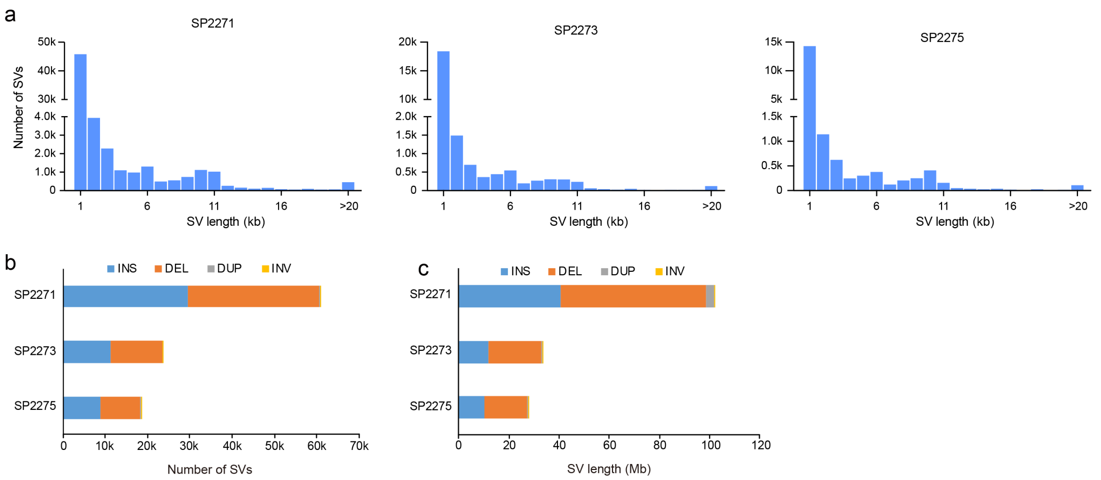

Supplementary Figure 7. Structural variations among *S. americanum* genomes. (a). SV length distribution. (b). Number of SVs in each accession. (c). SV length in each accession.

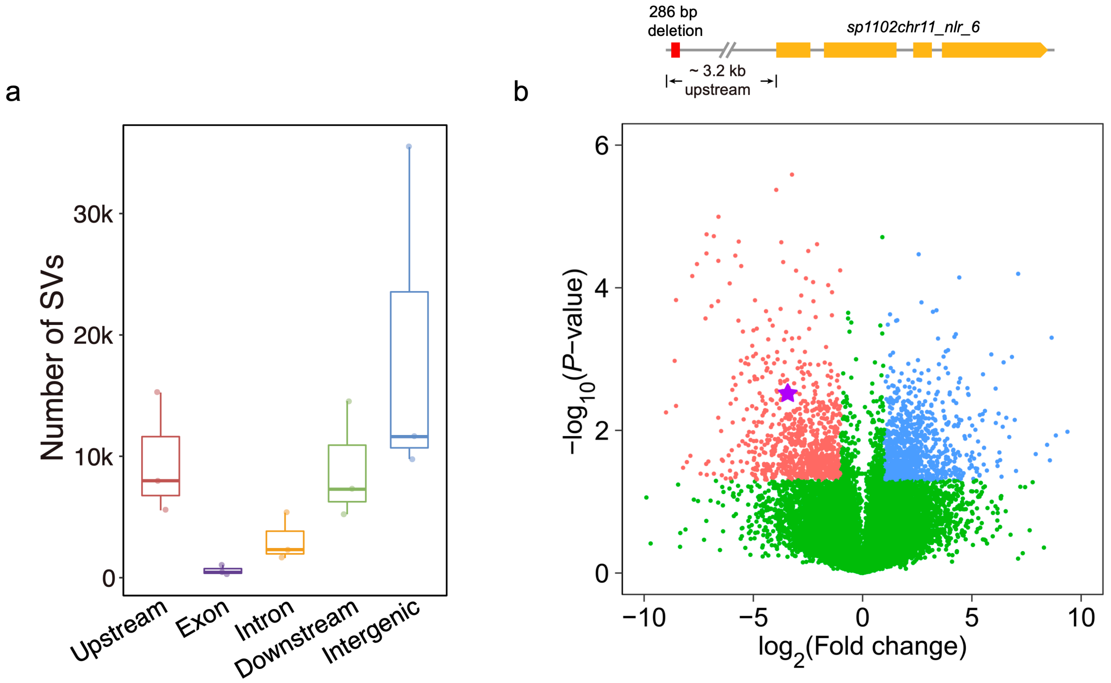

Supplementary Figure 8. SVs might contribute to gene expression variations. (a). Number of SVs overlapped with genomic features. Center line, median; box limits, upper and lower quartiles. The whiskers (top and bottom) comprise values within 1.5 times of the interquartile range (IQR). (b). An example of different expressed gene (DEG) whose expression might associate with the SV in its promoter region. Upper panel: The gene model of *sp1102chr11_nlr_6* with a deletion in its promoter region. Bottom panel: the volcano plot of DEGs in leaves between SP1102 and SP2271. Red and blue dots stand for DEGs (P-value < 0.05) with fold change <= 0.5 or >= 2, respectively; Green dots denote non-significantly expressed genes. The purple star: the expression of *sp1102chr11_nlr_6*. The stattest function in Ballgown package was used to perform statistical tests for differentially expressed genes using FPKM measurements. The function returned two-sided P-values and $\log_2$ fold changes for each gene.

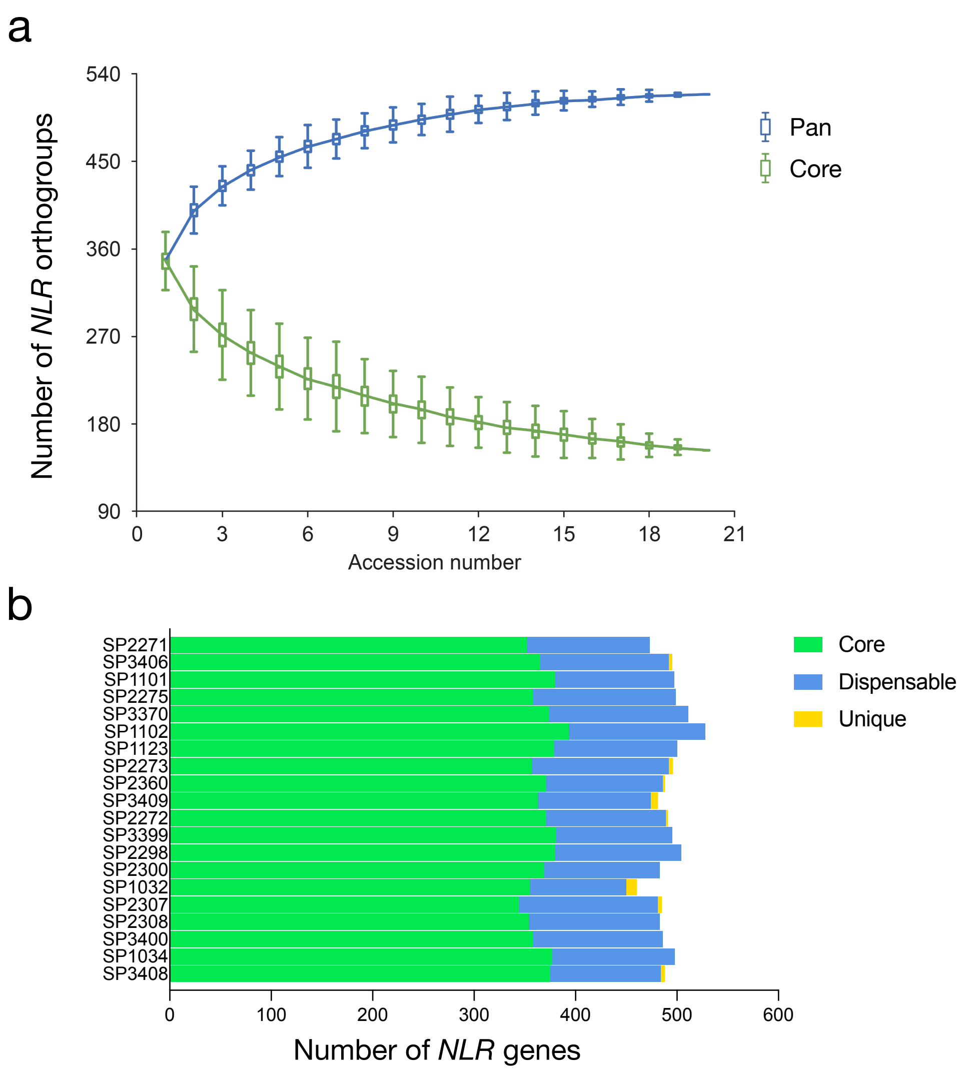

Supplementary Figure 9. Pan-NLRome of *S. americanum.* (a). Simulation of pan- and core-NLRome size of *S. americanum*. The NLR orthogroups were classified by OrthoFinder. The number in x axis indicates the number of *S. americanum* accessions were randomly selected for pan- and core-NLR analysis. For each accession number, 500 times of random selection with 30 replicates were performed. The estimated pan- and core-NLRome were fitted with exponential models. (b). Core, dispensable, and unique *NLR* genes from the 20 *S. americanum* accessions. Core *NLR* genes: *NLRs* present in all 18-20 accessions; Dispensable: *NLRs* were missed in more than 3 accessions and present in at least two accessions; Unique: *NLRs* present in only one accession.

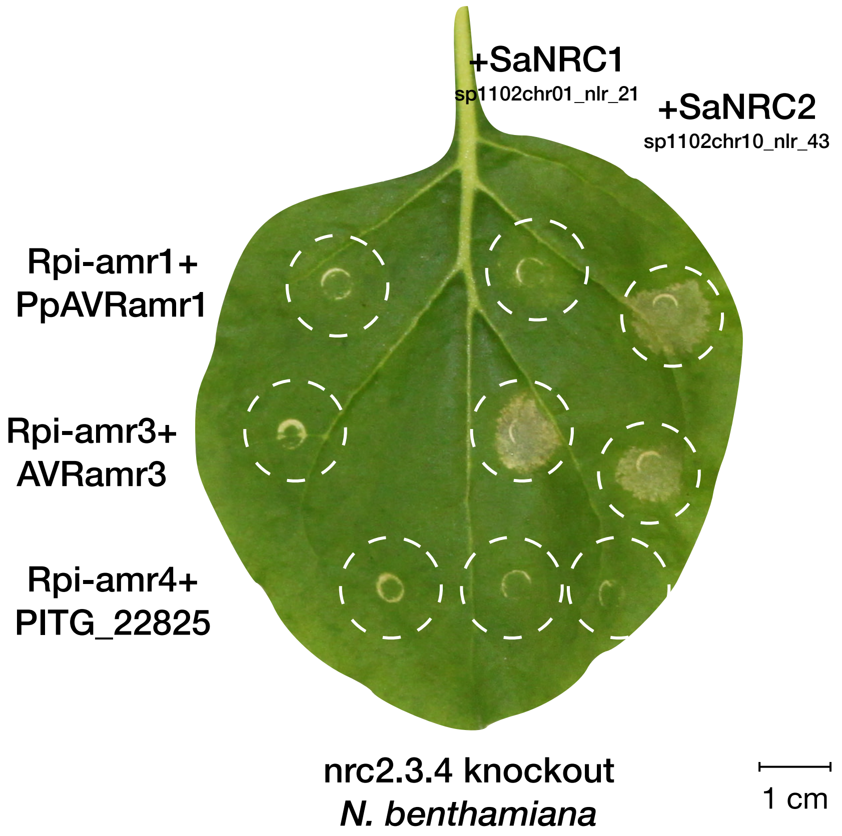

Supplementary Figure 10. SaNRC1 (sp1102chr01_nlr_21) supports the function of Rpi-amr3 but not Rpi-amr1 and Rpi-amr4-1102; SaNRC2 (sp1102chr10_nlr_43) supports the function of both Rpi-amr3 and Rpi-amr1, but not Rpi-amr4-1102. Rpi-amr1/PpAVRamr1; Rpi-amr3/AVRamr3; Rpi-amr4/AVRamr4 constructs were co-expressed, and co-expressed with SaNRC1 or SaNRC2 in nrc2.3.4 knockout *N. benthamiana*. OD_{600} = 0.5. Scale bar, 1 cm.

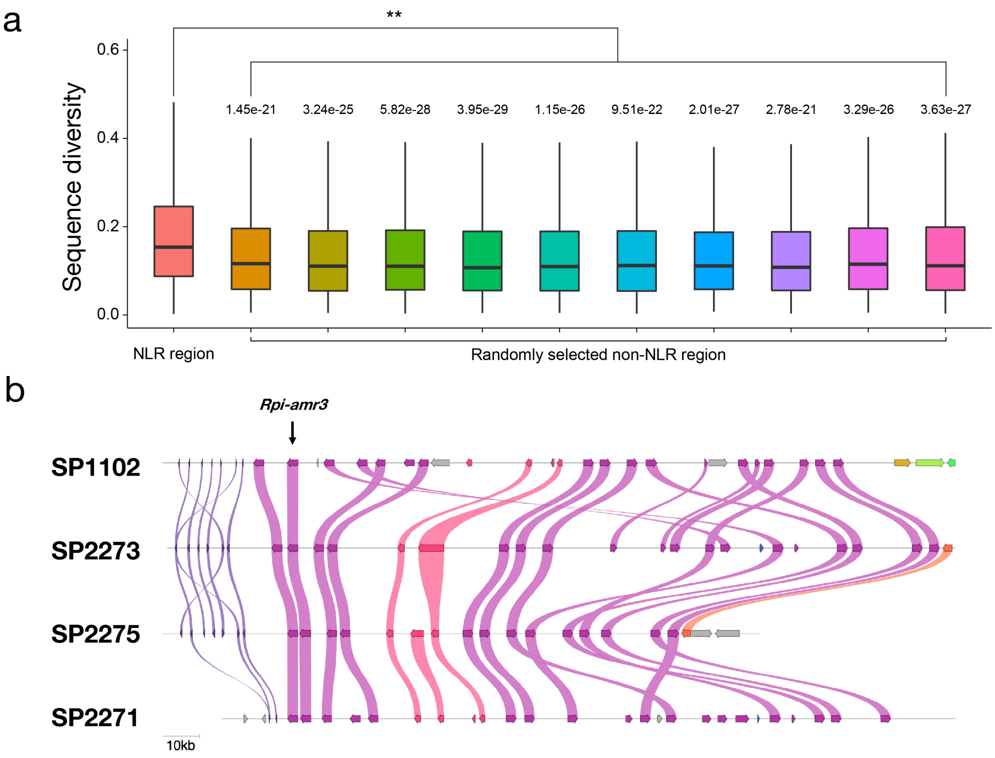

Supplementary Figure 11. Sequence diversity in NLR region. (a). The comparison of sequence diversity between NLR regions and non-NLR regions. Wilcoxon rank-sum test was used to assess the diversity values between NLR regions and non-NLR regions. The number above each box indicates two-sided *P*-value between NLR region and non-NLR region; Center line, median; box limits, upper and lower quartiles. whiskers (top and bottom) comprise values within 1.5 times of the interquartile range (IQR). ** stands for *P*-value < 0.01. (b). Synteny plot of *Rpi-amr3* locus indicates the sequence diversity of NLR regions. The color of arrows and links represent different gene families, it was clustered and visualized by clinker. All the *NLR* genes are purple, the genes with more than 0.3 identity were linked.

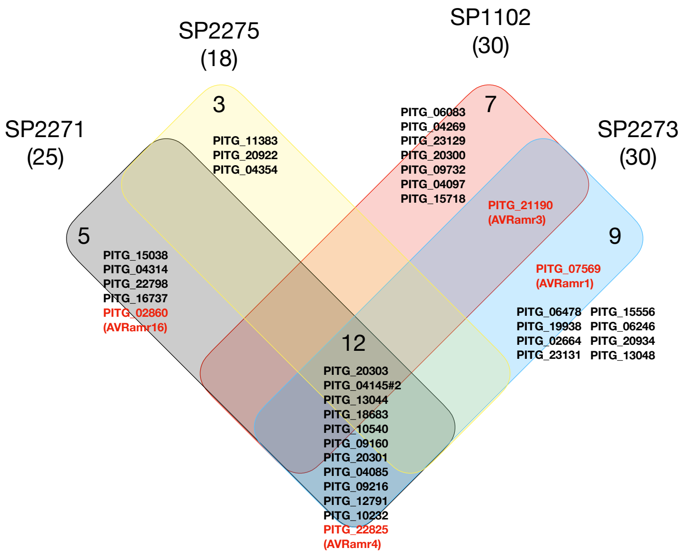

Supplementary Figure 12. Venn plot for effector recognition profiles in the four *S. americanum* accessions with reference genomes.

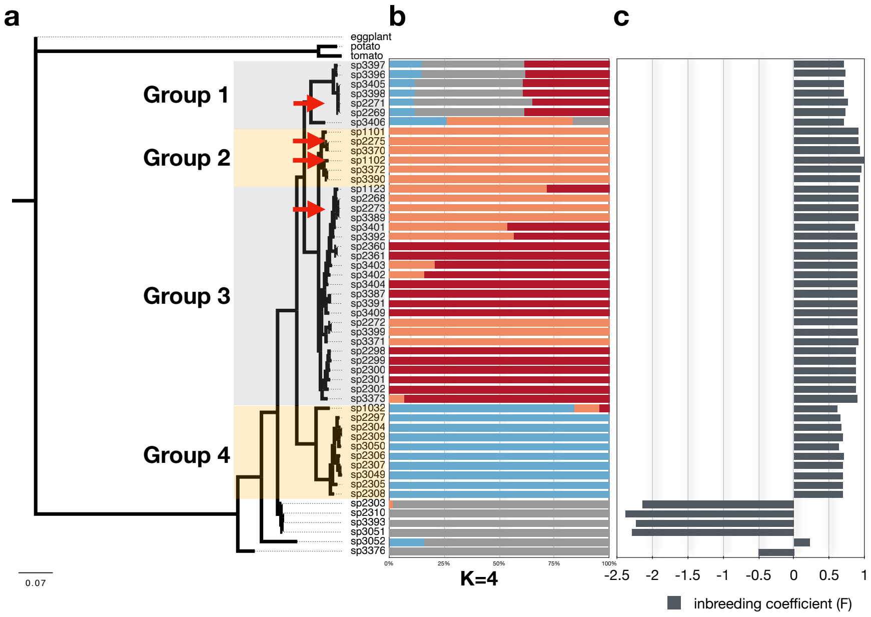

Supplementary Figure 13. Phylogeny, population structure and inbreeding coefficient of 52 *S. americanum* accessions. (a). The resequencing reads from the 52 *S. americanum* accessions were mapped to SP1102 genome, as well as the simulated reads from potato, tomato and eggplant genomes. Then all SNPs on genes were called and extracted, a maximum likelihood (ML) tree was inferred by IQ-TREE; (b). Structure analysis for 52 *S. americanum*, ADMIXTURE v1.3.0 was used for the analysis. All *S. americanum* SNPs on genes were used as inputs, K (number of subpopulations) =1, 2, 3, 4, 5, 6, 7 were calculated, and K= 4 is the best choice based on the cross validation (CV) errors. The population structure is present by the colour-coded stacked column chart. The colour reflects genetic contributions of their hypothetic ancestral populations. (c). The inbreeding coefficient (F) was calculated by VCFtools v0.1.14, all *S. americanum* SNPs on genes were used as inputs. Most accessions in group 1-4 have a high inbreeding coefficient value. The inbreeding coefficient value of some accessions are negative, indicates that these accessions are highly heterozygous, probably polyploid species.

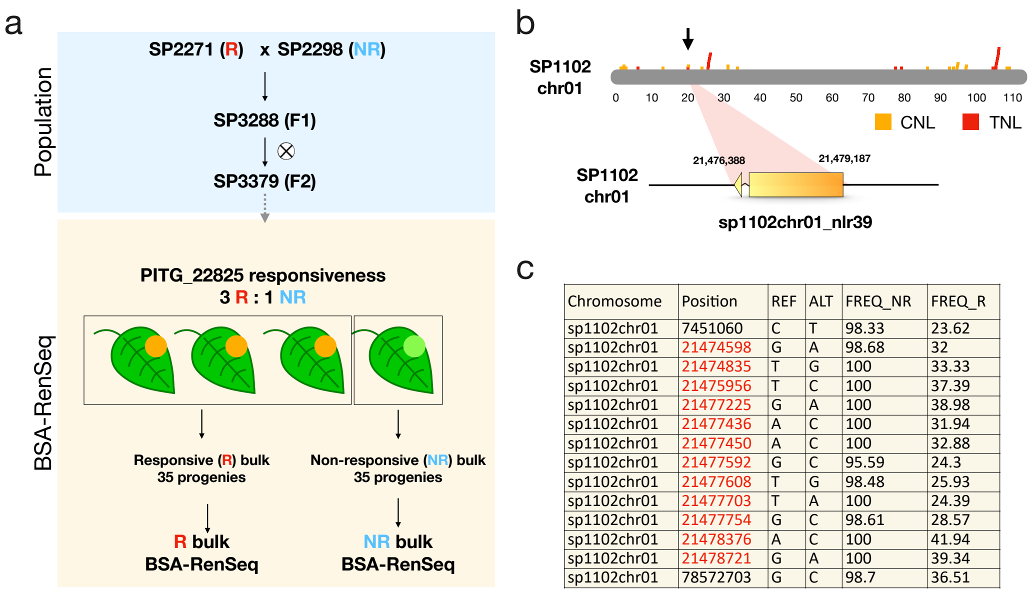

Chromosome	Position	REF	ALT	FREQ_NR	FREQ_R
sp1102chr01	7451060	C	T	98.33	23.62
sp1102chr01	21474598	G	A	98.68	32
sp1102chr01	21474835	T	G	100	33.33
sp1102chr01	21475956	T	C	100	37.39
sp1102chr01	21477225	G	A	100	38.98
sp1102chr01	21477436	A	C	100	31.94
sp1102chr01	21477450	A	C	100	32.88
sp1102chr01	21477592	G	C	95.59	24.3
sp1102chr01	21477608	T	G	98.48	25.93
sp1102chr01	21477703	T	A	100	24.39
sp1102chr01	21477754	G	C	98.61	28.57
sp1102chr01	21478376	A	C	100	41.94
sp1102chr01	21478721	G	A	100	39.34
sp1102chr01	78572703	G	C	98.7	36.51

Supplementary Figure 14. BSA-RenSeq for Rpi-amr4 in an F2 population of SP2271 x SP2298. (a). Pipeline of the BSA-RenSeq, leaf disks of the PITG_22825 responsive progenies, and non-responsive progenies were collected and pooled equally, then gDNA were extracted from both bulks. RenSeq library were prepared and sequenced. The reads were mapped to a *S. americanum* reference genome SP1102, then the SNPs were called and filtered. (b). Most filtered SNPs from the BSA-RenSeq are located on an *NLR* singleton sp1102chr01_nlr39. (c). The location of the filtered informative SNPs. REF: the reference SNP; ALT: alternative SNP; FREQ_NR, the alternative SNP frequency in the non-responsive bulk; FREQ_R: the alternative SNP frequency in the responsive bulk. The filtering criteria is 90%-100% FREQ_NR and 23%-43% FREQ_R.

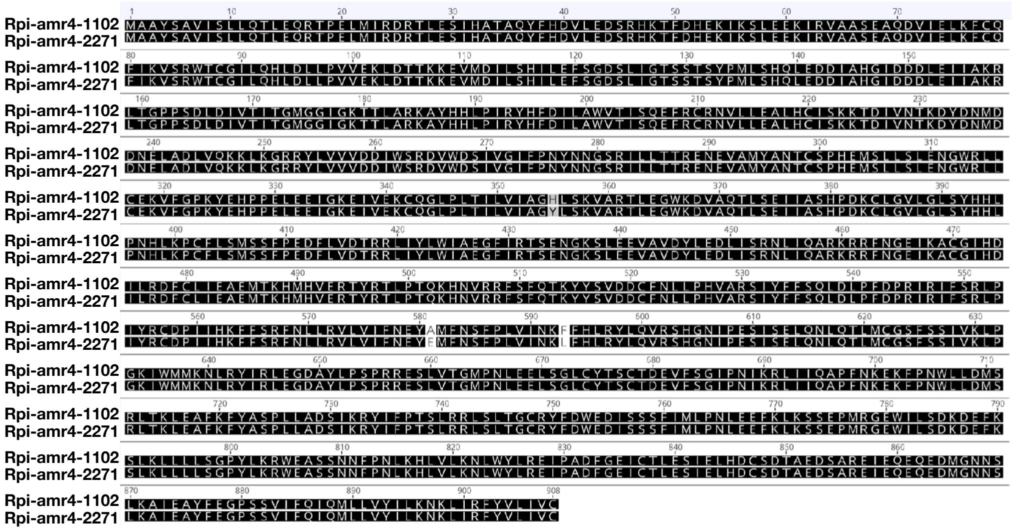

Supplementary Figure 15. Alignment of Rpi-amr4-1102 and Rpi-amr4-2271 proteins.

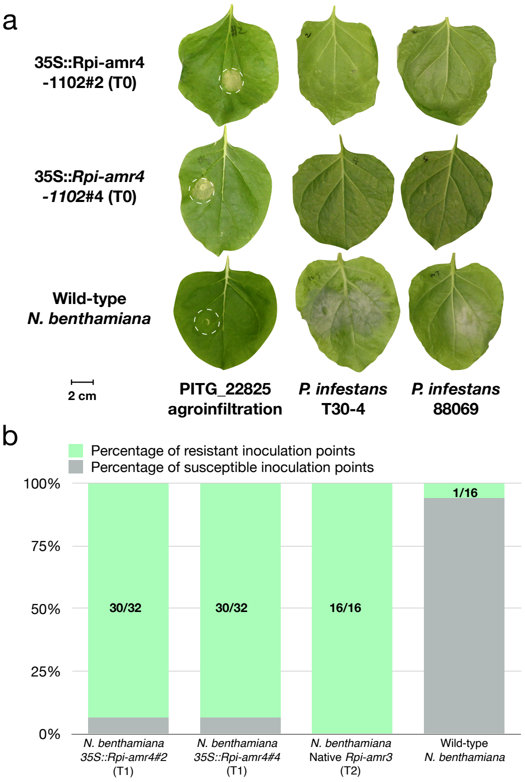

Supplementary Figure 16. *Rpi-amr4* confers late blight resistance in stably transgenic *N. benthamiana*. (a). PITG_22825 agroinfiltration and disease test on the *Rpi-amr4* transgenic *N. benthamiana*, two T0 lines (35S::*Rpi-amr4-1102*#2 and #4) were used in this assay. Wild-type *N. benthamiana* was used as a control. Scale bar, 2 cm. (b). The two T1 lines were used to verify this result, most inoculation points of the Rpi-amr4 transgenic plants are resistant to *P. infestans*. *P. infestans* T30-4 (300 zoospores/droplet) was used in both assays.

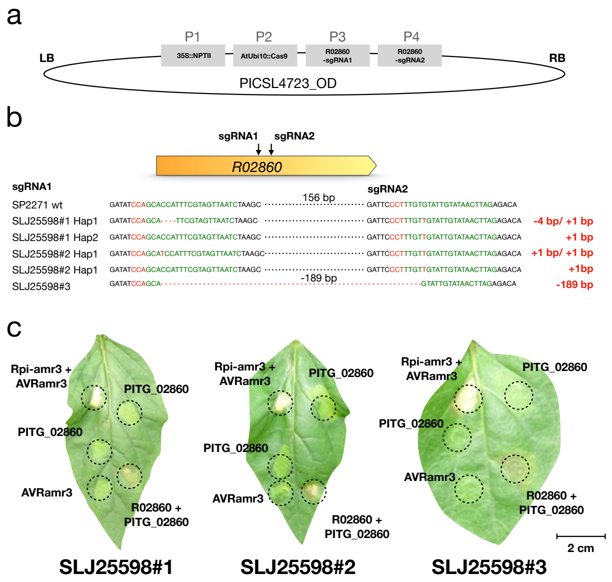

Supplementary Figure 17. *R02860* knockout SP2271 lines. (a). The constructs used for the CRISPR-Cas9 knockout experiment; (b). Genotyping data of three *R02860* lines SLJ25598#1, SLJ25598#2 and SLJ25598#3. (c). Phenotype of three selected knockout lines by agroinfiltration. Rpi-amr3 and AVRamr3 were used as controls. Scale bar, 2 cm.

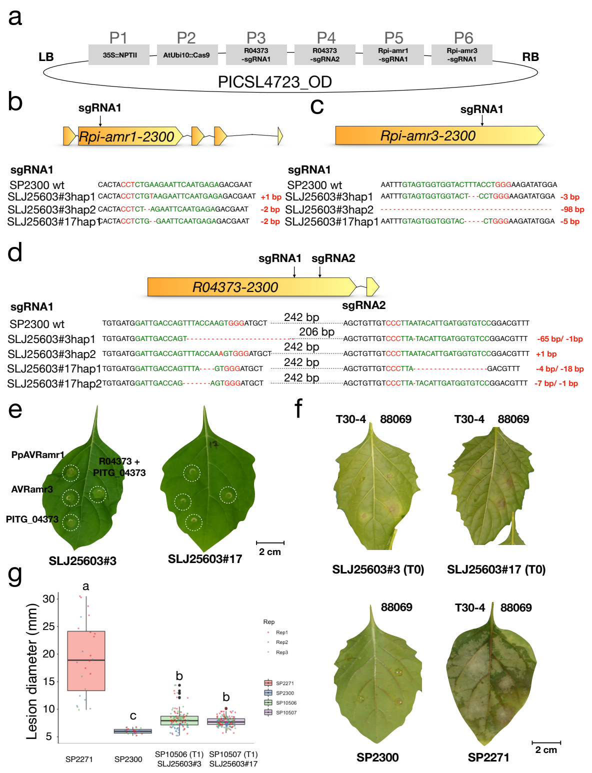

Supplementary Figure 18. *Rpi-amr1*/*Rpi-amr3*/*R04373* triple knockout SP2300 lines. (a). The constructs used for the CRISPR-Cas9 knockout experiment; (b, c and d). Genotyping the *Rpi-amr1-2300* and *Rpi-amr3-2300* and *R04373* in two knockout lines SLJ25603#3 and SLJ25603#17. (e). Phenotype of the triple knockout lines after expression of PpAVRamr1, AVRamr3 and PITG_04373. Co-expression of R04373 and PITG_04373 was used as a control. (f). Disease test on the two triple knockout lines (T0), *P. infestans* isolates T30-4 and 88069 was used in this assay. SP2300 and SP2271 were used as controls. (g). Disease test on the T1 plants of the two triple knockout lines were repeated and visualized. The zoospores (500 zoospores in a 10 µL droplet) from *P. infestans* strain T30-4 were used to inoculate the leaves. A calliper was used to measure the lesion size at 6 days after inoculation. Three biological replicates were performed, all datapoints were visualized as a box-and-whisker plot using R. Center line, median; box limits, upper and lower quartiles. The whiskers (top and bottom) comprise values within 1.5 times of the interquartile range (IQR). The outliers are indicated by black dots. Statistical differences were analysed by one-way ANOVA with Tukey's HSD test ($P < 0.001$). Scale bars, 2 cm.

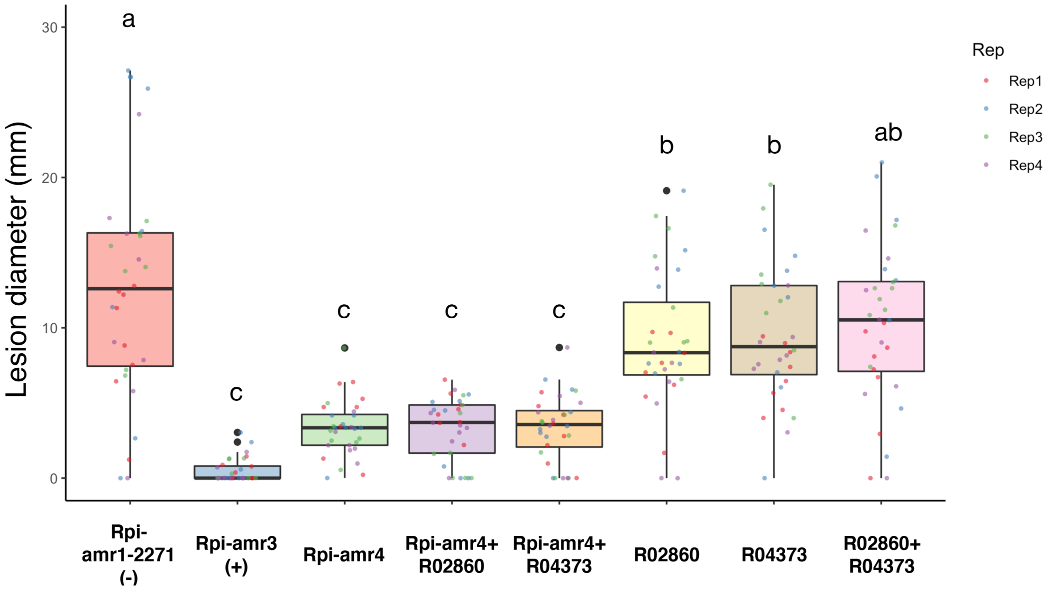

Supplementary Figure 19. Detached leaf assay (DLA) of 35S::*Rpi-amr4-1102*, R02860, R04373, and the combinations. Native *Rpi-amr3* (blue) and native *Rpi-am1-2271* (a non-functional *Rpi-amr1* paralogue, red) were used as positive and negative controls. All the constructs were transiently expressed in *N. benthamiana*, OD_{600} = 0.3. The zoospores (300 zoospores in a 10 µL droplet) from *P. infestans* strain T30-4 were used to inoculate the leaves 1 day after the infiltration. A calliper was used to measure the lesion size at 6 days after inoculation. Four biological replicates were performed, all datapoints (32 datapoints/treatment) were visualized as a box-and-whisker plot using R. Center line, median; box limits, upper and lower quartiles. The whiskers (top and bottom) comprise values within 1.5 times of the interquartile range (IQR). The outliers are indicated by black dots. Statistical differences were analysed by one-way ANOVA with Tukey's HSD test ($P < 0.001$).
