# Supplementary material for: Solanum americanum genome-assisted discovery of immune receptors that detect potato late blight pathogen effectors
Source: Nat Genet. 2023 Aug 28;55(9):1579–88. doi: 10.1038/s41588-023-01486-9 (PMC10484786; doi:10.1038/s41588-023-01486-9)
Supplement: Supplementary file 2 — Reporting Summary [file 41588_2023_1486_MOESM2_ESM.pdf]

nature portfolio

Corresponding author(s): Xiao Lin, Kee Hoon Sohn, Sanwen Huang, Jonathan D. G. Jones

Last updated by author(s): May 22, 2023

Reporting Summary

Nature Portfolio wishes to improve the reproducibility of the work that we publish. This form provides structure for consistency and transparency in reporting. For further information on Nature Portfolio policies, see our Editorial Policies and the Editorial Policy Checklist.

Statistics

For all statistical analyses, confirm that the following items are present in the figure legend, table legend, main text, or Methods section.

n/a	Confirmed	
☐	☒	The exact sample size (*n*) for each experimental group/condition, given as a discrete number and unit of measurement
☐	☒	A statement on whether measurements were taken from distinct samples or whether the same sample was measured repeatedly
☐	☒	The statistical test(s) used AND whether they are one- or two-sided<br>*Only common tests should be described solely by name; describe more complex techniques in the Methods section.*
☒	☐	A description of all covariates tested
☐	☒	A description of any assumptions or corrections, such as tests of normality and adjustment for multiple comparisons
☐	☒	A full description of the statistical parameters including central tendency (e.g. means) or other basic estimates (e.g. regression coefficient) AND variation (e.g. standard deviation) or associated estimates of uncertainty (e.g. confidence intervals)
☐	☒	For null hypothesis testing, the test statistic (e.g. *F*, *t*, *r*) with confidence intervals, effect sizes, degrees of freedom and *P* value noted<br>*Give P values as exact values whenever suitable.*
☒	☐	For Bayesian analysis, information on the choice of priors and Markov chain Monte Carlo settings
☒	☐	For hierarchical and complex designs, identification of the appropriate level for tests and full reporting of outcomes
☒	☐	Estimates of effect sizes (e.g. Cohen's *d*, Pearson's *r*), indicating how they were calculated

Our web collection on statistics for biologists contains articles on many of the points above.

Software and code

Policy information about availability of computer code

Data collection

No software was used for data collection.

Data analysis

KAT (v2.4.2), GenomeScope (v2.0), Hifiasm (v.0.13), juicer (v.1.5), 3d-DNA (v.180922), Canu (v1.8), SMARTdenovo (v8488de9), BWA (0.7.5a-r405), Pilon (v.1.23), BUSCO (), HISAT (v.2.0.1-beta), StringTie (v.1.3.3b), Cufflinks (v2.2.1), Trinity (v2.10.0), PASA (v2.4.1), SNAP (v.2013-02-16), AUGUSTUS (v.3.4.0), GlimmerHMM (v3.0.4), exonerate (v2.2), EVM (v1.1.1), GeMoMa (v1.7.1), OrthoFinder (v2.5.2), MAFFT (v.7.471), trimAl (v1.4.1), IQ-TREE (v2.1.4-beta), BASEML (v.4.9), MCMCTREE (v.4.9), MUMMER (v4.0.0rc1), D-Genies (v1.2.0), MCscan (Python version), Python (v3.5.6), minimap2 (v2.17-r941), SVIM-asm (v1.0.2), SnpEff (v5.0e), Geneious (v10.2.6), GMAP (v2020-10-14), STAR (2.6.0c), iTOL (v5), trimmomatic (v0.36), samtools (v1.9), bcftools (v1.9), Wgsim (2011 version), Bedtools (v2.17) , FigTree (v1.4.4), Plink (v1.90), R package qqman (v0.1.8), clinker (2020 version).

Some customized Python scripts were used to process the data generated by each software, which parameters were described in Methods section. All codes are available from the corresponding author upon request.

For manuscripts utilizing custom algorithms or software that are central to the research but not yet described in published literature, software must be made available to editors and reviewers. We strongly encourage code deposition in a community repository (e.g. GitHub). See the Nature Portfolio guidelines for submitting code & software for further information.

Data

Policy information about availability of data

All manuscripts must include a data availability statement. This statement should provide the following information, where applicable:

- Accession codes, unique identifiers, or web links for publicly available datasets
- A description of any restrictions on data availability
- For clinical datasets or third party data, please ensure that the statement adheres to our policy

The raw sequencing data for SP1102, SP2271, SP2273 and SP2275 genomes have been deposited at the National Center for Biotechnology Information (NCBI) Sequence Read Archive (SRA) with BioProject accession number PRJNA845062 (https://dataview.ncbi.nlm.nih.gov/object/PRJNA845062?reviewer=hliiufd2hm679172evsbdgcr69); The raw SMRT RenSeq data were deposit in ENA under project number: PRJEB38240; The whole genome resequencing data were deposit in ENA under project number: PRJEB57057; The BSA-RenSeq data were deposit in ENA under project number: PRJEB57070 and PRJEB57074. The assembled genomes, gene structure annotations, SMRT RenSeq assemblies, manually annotated NLR genes as well as variation information are available at Figshare (https://figshare.com/projects/The_Solanum_americanum_pangenome_and_effectoromics_reveals_new_resistance_genes_against_potato_late_blight/145449). The SaNRC1-1102, SaNRC2-1102, SaNRC3-1102, Rpi-amr4-1102, Rpi-amr4-2271, R02860 (Rpi-amr16) and R04373 (Rpi-amr17) sequences were deposited at NCBI GenBank under accession number: OP918030-OP918036.

Human research participants

Policy information about studies involving human research participants and Sex and Gender in Research.

Reporting on sex and gender	N/A
Population characteristics	N/A
Recruitment	N/A
Ethics oversight	N/A

Note that full information on the approval of the study protocol must also be provided in the manuscript.

Field-specific reporting

Please select the one below that is the best fit for your research. If you are not sure, read the appropriate sections before making your selection.

☒ Life sciences ☐ Behavioural & social sciences ☐ Ecological, evolutionary & environmental sciences

For a reference copy of the document with all sections, see nature.com/documents/nr-reporting-summary-flat.pdf

Life sciences study design

All studies must disclose on these points even when the disclosure is negative.

Sample size	No statistical analysis was used to determine the sample size. The sample size is widely used and accepted in the field, the details were described in the Methods section.
Data exclusions	For the disease assay in Figure 4d, the preliminary disease assay was not blinded and with a small sample size. The results were consistent with the 4 blind replicates, but the preliminary result was not included in the final statistical analysis and figure.
Replication	For Fig. 3: 3 biological replicates were performed for all the responsive effectors.<br>For the HR assays in Fig. 4 and Fig. 5: 3 biological replicates were performed.<br>For the disease assay in Figure 4d: 4 biological replicates were performed.<br>For the disease assay in Figure S19: 4 biological replicates were performed.
Randomization	All the plants in the same experiment were grew in the same condition. All samplings were randomized.
Blinding	For the disease assay in Figure 4d: Blind tests were performed for all the 4 replicates. For each replicate, a colleague streaked out the constructs (Rpi-amr3, Rpi-amr3a and Rpi-amr4) with a random code "A", "B" or "C". Another researcher performed the agroinfiltration, detached leaf assays, and scoring. The genotype codes were revealed after scoring.

Reporting for specific materials, systems and methods

We require information from authors about some types of materials, experimental systems and methods used in many studies. Here, indicate whether each material, system or method listed is relevant to your study. If you are not sure if a list item applies to your research, read the appropriate section before selecting a response.

Materials & experimental systems

n/a	Involved in the study
☒	☐ Antibodies
☒	☐ Eukaryotic cell lines
☒	☐ Palaeontology and archaeology
☒	☐ Animals and other organisms
☒	☐ Clinical data
☒	☐ Dual use research of concern

Methods

n/a	Involved in the study
☒	☐ ChIP-seq
☒	☐ Flow cytometry
☒	☐ MRI-based neuroimaging
